# Arterial Expression of the Calcium-Sensing Receptor Is Maintained by Physiological Pulsation and Protects against Calcification

**DOI:** 10.1371/journal.pone.0138833

**Published:** 2015-10-05

**Authors:** Guerman Molostvov, Thomas F. Hiemstra, Simon Fletcher, Rosemary Bland, Daniel Zehnder

**Affiliations:** 1 The Clinical Sciences Research Laboratory, Warwick Medical School, University of Warwick, Coventry, United Kingdom; 2 School of Clinical Medicine, University of Cambridge, Cambridge, United Kingdom; 3 Cambridge Clinical Trials Unit, Addenbrooke’s Hospital, Cambridge, United Kingdom; 4 Department of Nephrology, University Hospital Coventry and Warwickshire, Coventry, United Kingdom; University of Bari Aldo Moro, ITALY

## Abstract

Vascular calcification (VC) is common in chronic kidney disease (CKD) and contributes to cardiovascular mortality. The calcium-sensing receptor (CaSR) is present in human artery, senses extracellular calcium and may directly modulate VC. *Objective*: to investigate the association between arterial cyclic strain, CaSR expression and VC. *Methods and Results*: human aortic smooth muscle cells (HAoSMC) were cultured under static or strained conditions, with exposure to CaSR agonists, the calcimimetic R568, and after CaSR silencing and over-expression. High extracellular calcium reduced CaSR expression and promoted osteochondrogenic transformation and calcium deposition. This was partially prevented by cyclic strain and exposure to R568. CaSR silencing enhanced calcification and osteochondrogenic transformation, whereas CaSR over-expression attenuated this procalcific response, demonstrating a central role for the CaSR in the response to cyclic strain and regulation of VC. In arterial explants from CKD patients (n = 11) and controls (n = 9), exposure to R568 did not significantly alter calcium deposition, osteochondrogenic markers or total artery calcium content. *Conclusions*: physiological mechanical strain is important for arterial homeostasis and may protect arteries from VC. The beneficial effects of cyclic strain may be mediated via the CaSR.

## Introduction

The presence of arterial calcification is a powerful independent predictor of cardiovascular death in the general population [1] and particularly in patients with chronic kidney disease (CKD) [2,3]. Arterial calcification is present in almost 100% of patients with end-stage CKD [4,5]. In CKD disorders of mineral metabolism contribute to arterial calcification and diminished vascular compliance [6], leading to increased cardiac workload, heart failure, and sudden cardiac death [4].

Vascular calcification (VC) is a regulated, cell-mediated process similar to bone formation [7]. Several factors promote the transformation of contractile vascular smooth muscle cells (VSMC) to an osteoblast-like phenotype, including elevated calcium and phosphate concentrations, inflammatory mediators and uraemic toxins. Osteogenic transformation is characterised by increased expression of osteoblastic markers including bone specific alkaline phosphatase (ALP), osteocalcin (OC), runt-related transcription factor 2 (Runx2) and dentin matrix protein–1 (DMP–1) [8].

Normal vascular phenotype is maintained by pulsatile forces within the artery wall. VSMC express mechanoreceptors (integrins, receptor tyrosine kinases and ion channels) that sense and transduce pulsatile stretch to intracellular pathways, thereby modulating gene expression and cellular functions including proliferation, apoptosis and migration [9]. These functional changes result in vascular remodelling, which allows long-term adaptation to physiological and pathological conditions such as hypertension or metabolic stress [10–14].

The calcium-sensing receptor (CaSR) is a G protein-coupled cell surface receptor with wide tissue expression [15,16]. In the parathyroid gland it responds to changes in ionised calcium by regulating the synthesis and secretion of parathyroid hormone (PTH) [17]. Studies have identified the CaSR in cardiac [18] and artery tissue [19–21]. These data indicate that arteries, in particular VSMC sense and respond to extracellular calcium directly. We have previously shown an association between CaSR expression and medial artery calcification [21,22]. In addition, *in vitro* experiments support a potential role for the CaSR in the regulation of VC [23,24]. The emergence of calcimimetic agents which, unlike calcium, do not directly contribute to calcification, provided strong evidence for the importance of the CaSR in vascular calcification [23–25]. Calcimimetics, which allosterically activate the CaSR, have proved effective in lowering PTH levels without increasing serum calcium and phosphorus concentrations [26,27] and are in wide clinical use. In addition, calcimimetics may exert a direct effect on the vessel wall protecting it from vascular calcification. [5,24,28]. *In vitro*, CaSR stimulation with calcimimetics inhibits calcium- and phosphate-induced mineralisation [23,24,28]. *In vivo* evidence from both animal [29] and human studies [5,30,31] suggests that calcimimetics attenuate vascular and valvular calcification. Evidence from a recent large interventional outcome trial suggests that systemic targeting of the CaSR may translate into lower cardiovascular event rates in CKD patients [31].

In this study, we aimed to determine the effect of cyclic strain on the phenotype and response to calcifying stimuli of VSMC, and to establish to what extent these effects were mediated by the CaSR.

## Materials and Methods

### Cell culture

Primary cultures of human aortic smooth muscle cells (HAoSMC) (PromoCell) were maintained in complete VSMC growth medium 2 as described previously [22]. For cyclic strain culture, cells were plated into 6-well collagen 1 coated Bioflex plates (Flexcell) and cultured under cyclic biaxial strain for up to 14 days using Flexcell FX–4000 unit. 7% stretch was chosen to model artery pulsatile wall stretch [32]. Frequency of 30 cycles/min allowed cells to remain attached to the Bioflex plate for 7 days. For calcification experiments, cells were incubated for 7 days with 2 and 5mmol/L Ca^2+^, 50μmol/L Gd^3+^ or with combination of 2mmol/L Ca^2+^ and 50μmol/L Gd^3+^. Alternatively, HAoSMC were treated with 2 and 5mmol/L Ca^2+^ in the presence of 10, 100, 1000nmol/L calcimimetic R563 or 1000nmol/L S568 (inactive enantiomer) (Amgen). Control group was treated with 1.1 mmol/L Ca^2+^. To facilitate mineralisation, 5mmol/L -glycerophosphate (-GP) was added to all calcification experiments.

Since pilot experiments (S1 Fig) had demonstrated HAoSMC phenotypic stability over at least 7 days, we used a 7 day time period for all cell culture experiments.

### Human arterial explant culture

Human artery collection was performed at the University Hospital Coventry and Warwickshire NHS Trust, UK after obtaining written informed consent. Ethical approval was obtained from Coventry Research Ethics Committee (05/Q2802/26), UK. Fresh surgically removed human renal and epigastric arteries from 9 healthy kidney donors (control) and 11 patients with end-stage CKD undergoing renal transplant (CKD) (Table 1) were cut into small rings (approx. 2 mm in length and 2–3 mm in diameter). They were equilibrated and washed for 1 hour in plain VSMC growth medium. Arterial explants were cultured in complete VSMC growth medium 2 for 7 days and treated with 5mmol/L Ca^2+^ with or without 100nmol/L R568 or S568. Following treatment arterial rings were washed and snap frozen in liquid nitrogen. Further, the samples were mechanically ground and homogenised in liquid nitrogen and re-suspended in RIPA buffer for Western blotting as previously described or specific buffers for Runx2 and DMP–1 ELISA supplied with respective ELISA kits (MyBioSource).

**Table 1:** Clinical characteristics of the patients donating medium sized artery.

Variables	CKD	Control	P value
**N**	11	9	
**Age, years**	47.5 (20–75)	65.5 (39–79)	0.09
**Male, n (%)**	6 (60)	7 (70)	
**Ethnicity, caucasian / asian**	8/2	10/0	
**BMI, kg/m** ^**2**^	24.1 ± 3.5	27.5 ± 2.1	0.01
**Smoking ever, n (%)**	2 (20)	2 (20)	
**Hypertension, n (%)**	9 (90)	3 (30)	
**Systolic BP, mm Hg**	135.0 ± 24.4	124.0 ± 11.1	0.34
**Diastolic BP, mm Hg**	85.0 ± 11.7	74.9 ± 8.3	0.04
**Diabetes mellitus, n (%)**	1 (10)	0	
**Dialysis, n (%)**	8 (80)	0	
**Dialysis vintage, months**	12.7 ± 15.6	0	
**Creatinine, mg/dl**	6.8 ± 2.4	0.9 ± 0.3	<0.001
**eGFR, ml/min/1.73m** ^**2**^	-	88.4 ± 31.4	
**Hemoglobin, g/dl**	11.7 ± 1.1	14.5 ± 2.2	0.02
**Calcium, mg/dl**	9.1 (8.6–9.6)	8.9 (8.4–9.2)	0.33
**Phosphate, mg/dl**	4.9 (1.1–2.2)	2.9 (1.7–3.4)	<0.001

Data are mean ± SD, median (range) or frequencies (%). P value—by paired-samples t-test. BMI, body mass index; BP, blood pressure; eGFR, estimated glomerular filtration rate; CKD, patients with end-stage chronic kidney disease; Control, donors with maintained renal function.

### Inhibition of CaSR expression by specific siRNA

Transfection of CaSR siRNA (Santa Cruz Biotechnology) into HAoSMC was performed using lipofectamine (Invitrogen) according to manufacturer’s protocol. Control siRNA-A (Santa Cruz) and lipofectamine alone were used as negative controls. To maintain inhibition of the CaSR expression in longer-term cultures, transfection was repeated every 3 days. CaSR knockdown was monitored by Western blot. Following transfection the cells were cultured for 7 days and treated with 2 and 5mmol/L Ca^2+^, 50mol/L Gd^3+^ or in combination.

### Over-expression of the CaSR by pcDNA3.1CaSR+

For transfection, full length human CaSR was cloned into pcDNA3.1(+) containing hygromycin selection gene. Plasmid DNA was prepared using Qiagen plasmid midi kit (Qiagen). HAoSMC were seeded at 2×10^5^ cells/well of 6-well plate, allowed to adhere overnight, and washed twice with OptiMEM medium (Invitrogen). Transfection of pcDNA3.1CaSR+ into HAoSMC was performed using Lipofectamine LTX with Plus Reagent (Invitogen) according to manufacturer's protocol. Lipofectamine and plasmid DNA were diluted into OptiMEM. Diluted lipofectamine lipids were mixed with diluted plasmid DNA and incubated for 30 min at room temperature for complex formation. Mixtures were further diluted in OptiMEM, carefully added to each well to make final concentration of 2μg/ml and incubated overnight. After 24 hour rest in complete medium, the cells were cultured for 72 hours in selection medium containing 100μg/ml hygromycin (Sigma). Control (empty) pcDNA3.1(+) and lipofectamine alone were used as negative controls. The effectiveness of transfection (CaSR over-expression) was monitored by RT-PCR and Western blotting. After transfection HAoSMC were cultured for 7 days and treated with 2 and 5mmol/L Ca^2+^, 50mol/L Gd^3+^ or in combination.

### Analysis of the CaSR mRNA expression

Total RNA was isolated from HAoSMC lysates using RNA easy kit (Qiagen) following the manufacturer's protocol. Reverse transcription was carried out using a Bioscript reverse transcriptase with random hexamers (Bioline). PCR amplification of CaSR cDNA was performed as described previously [22].

### Western blot analysis

HAoSMC and arterial explants were treated with specified agonists and harvested as described previously [22]. Cell lysates were separated by SDS-PAGE and Western blotted with anti-β-actin (New England Biolabs), anti-CaSR (Binding Site), anti-Runx2 (Santa Cruz Biotechnology). Densitometry was performed using Image J Analysis software using blots from four independent experiments with final results normalised against β-actin.

### Alizarin red staining

HAoSMC were rinsed with 0.9% NaCl solution (pH = 7.4), stained with 1% alizarin red (pH = 4.2) for 1 min and further rinsed twice with 0.9% NaCl. Cell cultures were photographed using Nikon TS100 System. The percentage of alizarin red-positive areas was measured using Image J analysis software.

### Analysis of osteoblast-like phenotype

OC production was assayed using N-MID OC ELISA (IDS) following the manufacturer’s protocol (CV<4%). The cells were harvested, solubilised with RIPA buffer and assayed using N-MID OC ELISA. ALP activity was measured using SensoLyte p-Nitrophenyl phosphate ALP kit (AnaSpec). Following treatment the cells were scraped, solubilised and assayed. Runx2 and DMP–1 (CV<8%) in arterial explants were quantified with ELISA kits (MyBioSource). Arterial tissue was homogenised, lysed and assayed for protein concentration. OC, ALP, Runx2 and DMP–1 expression (ng/ml) was normalised against protein concentration of the samples.

### Calcium content measurement

Calcium was measured by a colorimetric assay using orthocresolphthalein complexone method developed by Schwartzenbach and modified by Connerty & Briggs. Arterial explants were homogenised, lysed and assayed for protein concentration. The extracts were acidified with 50% HCl and briefly centrifuged before analysis. The absorbance was read at 550nm using Victor3 plate reader (Perkin Elmer). Calcium content (mmol/L) was normalised against protein concentration of the samples.

### Data analysis

The number of replicates for each experiment is provided in the legend of the relevant figure. Data are represented in text as means and 95% confidence intervals (95%CI) unless specified as median (interquartile range (IQR)). These data are graphically represented as box plots, showing the sample median and IQR, with whiskers representing the nearest adjacent (the nearest value up to, but not exceeding, 1.5 times the IQR from the median). Given that the distribution of most variables described here is known to be normal, and that sample sizes are relatively modest, comparisons of means were made by two-sample Student’s T-test (unless otherwise specified), with no adjustment for multiple comparisons. For comparisons of arterial variables by CKD status across experimental conditions, groups were compared using one-way ANOVA. Statistical significance was arbitrarily set at p<0.05, although precise type-I errors are reported throughout. All analyses were carried out using Stata SE 13.1 (College Station, TX).

## Results

### Cyclic strain inhibits calcium deposition and maintains CaSR expression by HAoSMC

To assess whether exposure of HAoSMC to CaSR agonists impacted on their propensity to calcify, HAoSMC were cultured under control conditions and upon exposure to calcium and the non-calcium CaSR agonist, gadolinium, as follows: Control (Ca^2+^ 1.1mmol/L), moderate (Ca^2+^ 2.0mmol/L), and high calcium (5mmol/L), hereafter denoted *Control*, *Ca2* and *Ca5* respectively; and Gadolinium 0.05mmol/L with either Ca^2+^ 1.1mmol/L or Ca^2+^ 2.0mmol/L, denoted *Gd* and *Gd+Ca2* respectively. All values are reported as Alizarin red positive area (%).

**Fig 1:**
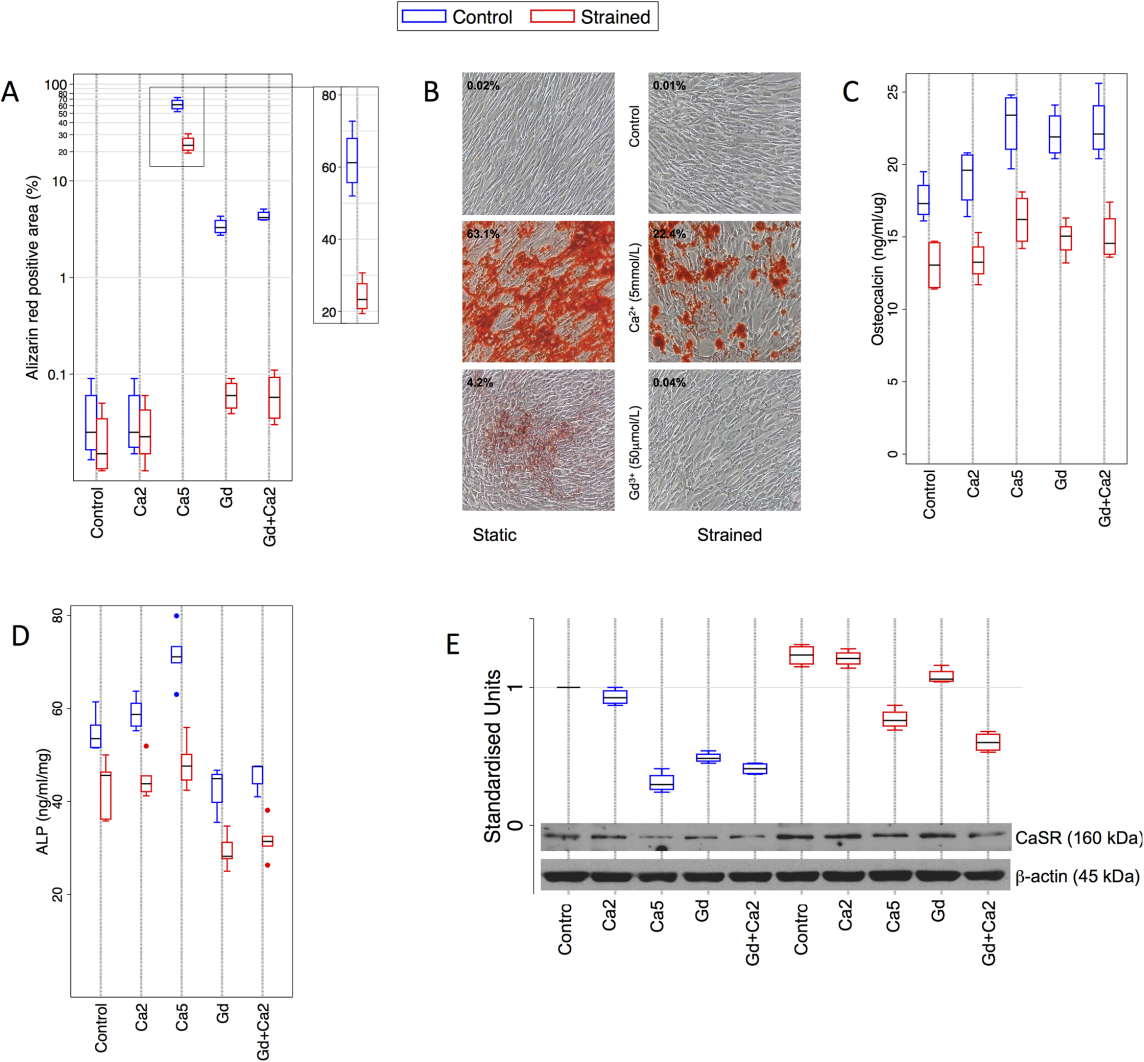
Effect of cyclic biaxial strain and CaSR agonists on HAoSMC CaSR expression and phenotype. **A)** Calcium deposition was measured with alizarin red staining (n = 4) for each condition. Data are represented on a logarithmic y-axis. Exposure to CaSR agonists (*Ca5*, *Gd*, *Gd2+Ca5*) significantly increased calcification, and this was reduced by cyclic strain. **B)** Representative photographs of alizarin red staining (magnification x100). **C)** OC expression was determined by ELISA, and corrected for protein concentration (n = 4 for each condition). In static HAoSMC, OC expression was significantly increased by *Ca5*, *Gd*, and *Gd+Ca2*. In comparison, cyclic strain significantly reduced OC expression compared to static cells under all conditions (*Control* p = 0.008; *Ca2* p = 0.004; *Ca5* p = 0.004; *Gd* p = 0.0004; *Gd+Ca2* p = 0.002). **D)** ALP activity (n = 5 for each condition) increased with *Ca5* in static cells, but decreased in the presence of Gadolinium. Under all conditions, cyclic strain reduced ALP activity compared to static HAoSMC (*Control* p = 0.008; *Ca2* p = 0.0004; *Ca5* p = 0.004; *Gd* p = 0.0002; *Gd+Ca2* p = 0.001). **E)** CaSR protein expression (n = 4 for each condition) was reduced by *Ca5*, *Gd* and *GD+Ca2*, whereas cyclic strain increased CaSR expression and attenuated CaSR agonist-induced reductions in CaSR expression. OC–osteocalcin; ALP–alkaline phosphatase; CaSR–calcium-sensing receptor.

*Control* HAoSMC demonstrated virtually no calcification (0.38%, 95%CI 0–0.9%) after 7 days incubation. *Ca2* HAoSMC did not differ from control (0.39%, 95%CI 0–0.9%, p = 0.98) (Fig 1A and 1B). In contrast, *Ca5* HAoSMC demonstrated marked calcification (61.8%, 95%CI 48–75.5%, p<0.0001). Significant calcification also occurred in *Gd* (3.4%, 95%CI 2.3–4.5%, p = 0.0001) and *Gd+Ca2* HAoSMC (4.3%, 95%CI 3.5–5.2%, p<0.0001), but to a much lesser extent than with *Ca5*, suggesting a role for the CaSR in modulating calcification *in vitro*.

Since aortic smooth muscle cells are exposed to cyclic strain *in vivo*, we next determined whether HAoSMC exposed to cyclic strain (hereafter sHAoSMC) responded similarly to CaSR agonists. Surprisingly, we noted a considerable reduction in calcification: *Ca5* sHAoSMC exhibited a highly significant 60% reduction (24.1%, 95%CI 16.5–31.8%, p = 0.0003) in calcification compared to unstrained *Ca5* cells. In *Gd* (0.6%, 95%CI 0.03–0.1%) and *Gd+Ca2* (0.06%, 95%CI 0.01–0.12%) sHAoSMC, calcification was similarly reduced by 82% (Gd, p = 0.0001) and 86% (Gd+Ca2, p<0.0001) respectively compared to unstrained cells.

To establish whether the inhibition of calcification observed in sHAoSMC was associated with differential expression of protein markers of osteogenic phenotype transformation, we determined the concentration of OC and ALP activity in static and strained cells. Under control conditions, OC concentration was 17.5 (95%CI 15.3–19.8) ng/ml per mg protein. OC was significantly increased by the addition of *Ca5* (22.8, 95%CI 19.1–26.5, p = 0.009), *Gd* (22.1, 95%CI 19.5–24.7, p = 0.006) and *Gd+Ca2* (22.6, 95%CI 19.0–26.1, p = 0.009). These changes mirrored the extent of calcification observed earlier. In contrast, cyclic strain reduced OC expression compared to matched unstrained HAoSMC under all conditions (p<0.01, Fig 1C).

ALP activity was increased by exposure to *Ca5* in static HAoSMC (71, 95%CI 64–79 ng/ml) compared to *control* (55, 95%CI 50–60, p = 0.001). Interestingly however, *Gd* exerted a differential effect: ALP activity was reduced by *Gd* (43, 95%CI 37–48, p = 0.002) and *Gd+Ca2* (45, 95%CI 42–49, p = 0.003). Similar to OC, we observed reduced ALP activity in sHAoSMC under all conditions when compared to matched static cells (p<0.01, Fig 1D). Furthermore, under cyclic strain, HAoSMC were protected from *Ca5-*induced increases in ALP activity.

Given these observations, we asked whether these stimuli modulated protein expression of the CaSR in HAoSMC. Data were normalised to mean CaSR expression in unstrained cells (Fig 1E). Exposure of static HAoSMC to *Ca5* (0.31, 95%CI 0.19–0.43, p<0.0001), *Gd* (0.49, 95%CI 0.43–0.55, p<0.0001) and *Gd+Ca2* (0.41, 95%CI 0.35–0.47, p<0.0001) profoundly reduced CaSR expression compared to control HAoSMC. In contrast, cyclic strain increased CaSR expression in *control* HAoSMC compared to static cells (1.23, 95%CI 1.11–1.35, p = 0.0008), and appeared to attenuate the reduction in CaSR expression observed with CaSR agonists in unstrained cells. However, significant reductions were still observed with *Ca5* (0.77, 95%CI 0.65–0.89, p = 0.0008) and *Gd+Ca2* (0.6, 95%CI 0.49–0.71, p<0.0001).

### Reduced expression of the CaSR increases calcium deposition by HAoSMC

**Fig 2:**
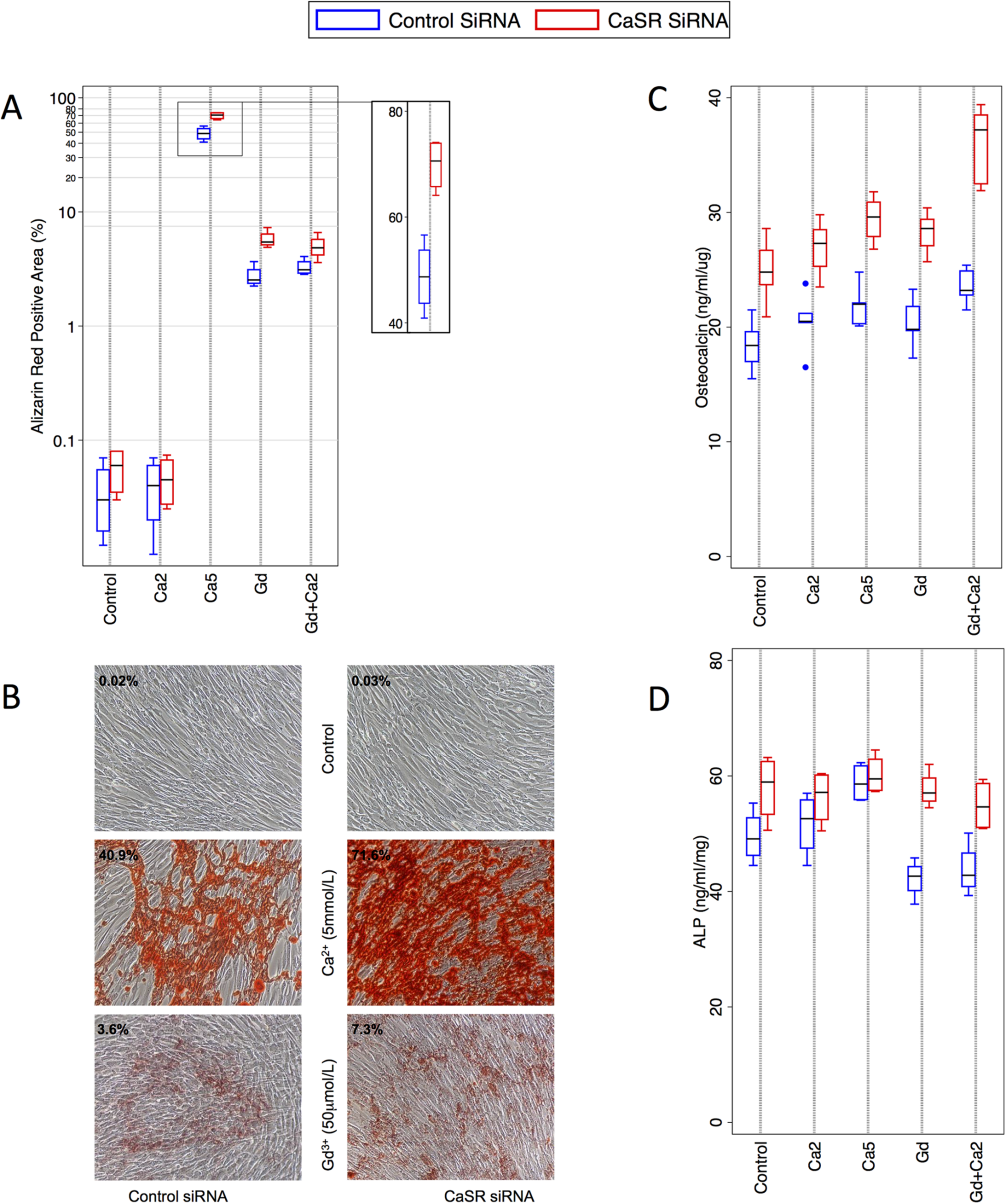
Effect of CaSR silencing on HAoSMC calcification and phenotype. **A)** Calcium deposition (measured by alizarin red, n = 4 per condition, logarithmic y-axis) was significantly higher after CaSR silencing in *Ca5*, *Gd* and *Gd2+Ca2* HAoSMC. **B)** Representative photographs of alizarin red staining (magnification x100). **C)** OC expression (n = 5 per condition) was significantly increased by CaSR knockdown across all conditions (*Control* p = 0.004; *Ca2* p = 0.004; *Ca5* p = 0.0003; *Gd* p = 0.0003; *Gd+Ca2* p = 0.0001). **D)** ALP activity (n = 4 per condition) was significantly higher with CaSR silencing compared to control in the presence of Gadolinium (*Gd*, p = 0.0005; *Gd+Ca2*, p = 0.01). OC–osteocalcin; ALP–alkaline phosphatase; CaSR–calcium-sensing receptor.

To determine whether HAoSMC calcification was dependent upon CaSR expression, we first compared calcium deposition in HAoSMC after CaSR silencing to that observed under control conditions. We had previously demonstrated that CaSR knockdown resulted in 60–80% decrease in CaSR expression in HAoSMC over 7 days [21,22], and employed the same approach. Consistent with our earlier findings (above), calcification was virtually absent in *control* and *Ca2* HAoSMC, and this was not altered by CaSR silencing. However, under conditions we have previously observed significant calcium deposition (*Ca5*, *Gd*, *Gd+Ca2*), CaSR silencing resulted in further significant increases in calcium deposition. *Ca5* CaSR knockdown cells exhibited 70% calcification (95%CI 62–78), increased from 49% (95%CI 38–59) in *Ca5* controls (p = 0.002), whereas both *Gd* CaSR knockdown (5.8, 95%CI 4.1–7.4 versus 2.7, 95%CI 1.7–3.8, p = 0.003) and *Gd+Ca2* CaSR knockdown (5.0, 95%CI 3.0–6.9 versus 3.3, 95%CI 2.4–4.1, p = 0.046) HAoSMC also demonstrated significant but lesser increases in calcium deposition (Fig 2A and 2B).

We next assessed the effects of CaSR silencing on OC and ALP. Across all conditions, OC concentrations were significantly increased by CaSR knockdown (p<0.004, Fig 2C). ALP activity was increased in the presence of Ca5 although this did not reach statistical significance (Fig 2D). In contrast, in the presence of Gd (as noted before, Fig 1D), ALP activity was reduced. However, CaSR knockdown abolished the *Gd* (p = 0.0005) and *Gd+Ca2* (p = 0.013) associated reduction in ALP activity. These findings suggested that reduced CaSR expression resulted in increased osteochondrogenic differentiation of HAoSMC. Further, we observed that the differential effects of *Ca5* versus *Gd*/*Gd+Ca2* on ALP activity (Fig 1D) were abolished with CaSR silencing, indicating that changes in ALP expression were CaSR dependent (Fig 2D).

### CaSR over-expression reduces calcium deposition by HAoSMC

**Fig 3:**
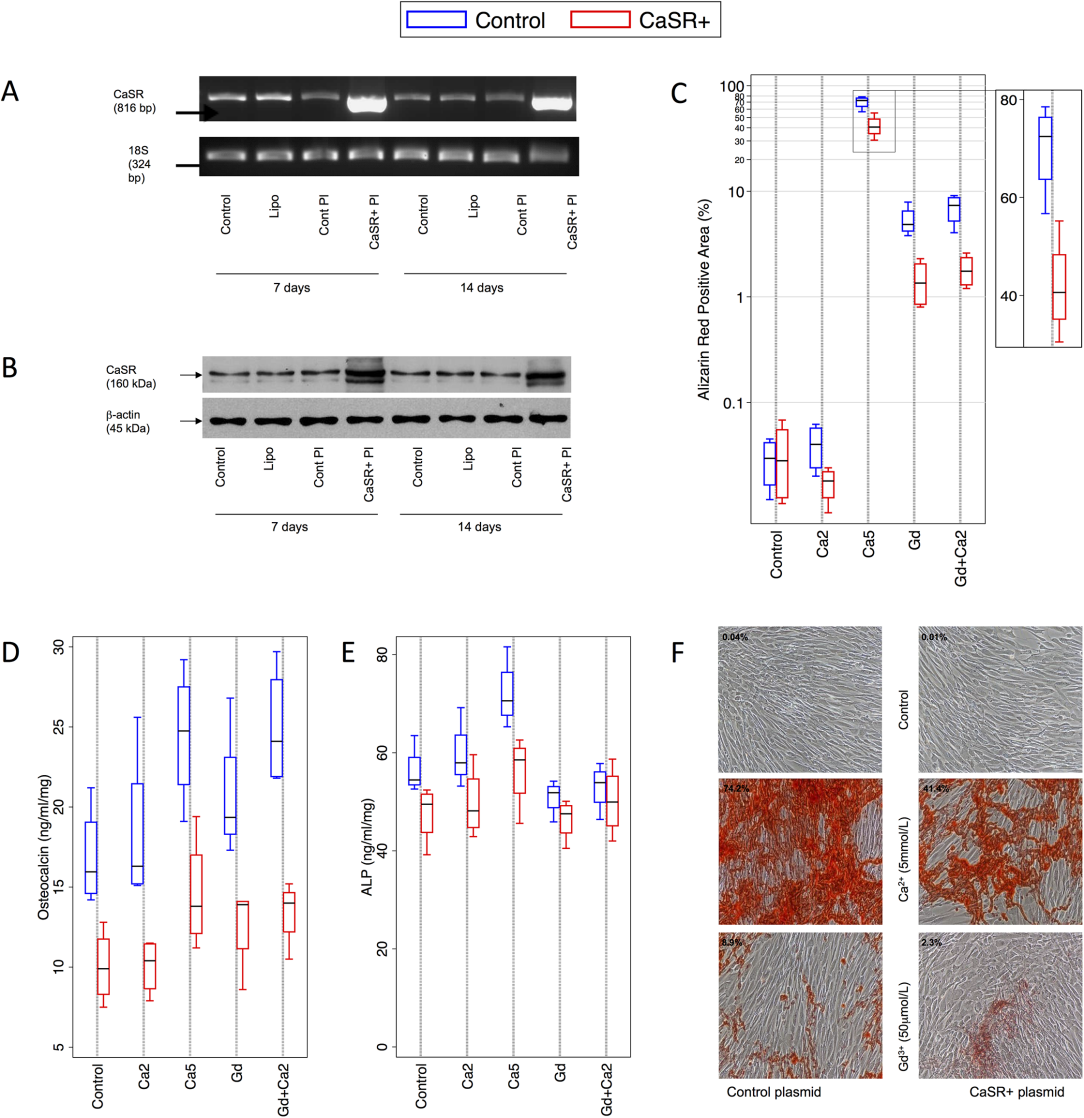
Effect of CaSR over-expression and CaSR agonists on HAoSMC calcification and phenotype. HAoSMC were transfected with pcDNA 3.1 CaSR+. **A)** An increase in CaSR mRNA and **B)** CaSR protein expression in HAoSMC transfected with pcDNA 3.1 CaSR+ after 7 and 14 day culture. **C)** Calcium deposition (measured by alizarin red, n = 4 per condition, logarithmic y-axis) was significantly lower after CaSR over-expression compared to controls in *Ca5*, *Gd* and *Gd2+Ca2* HAoSMC. **F)** Representative photographs of alizarin red staining (magnification x100). **D)** OC expression (n = 4 per condition) was markedly lower with CaSR over-expression across all conditions. **E)** ALP activity (n = 4 per condition) was lower overall with CaSR over-expression, and significantly lower than the respective control in the presence of *Ca5*. OC–osteocalcin; ALP–alkaline phosphatase; CaSR–calcium-sensing receptor.

To further corroborate these findings, HAoSMC were transfected with pcDNA3.1CaSR+ to over-express the CaSR, resulting in a 4.5 to 5-fold increase in CaSR mRNA sustained for 14 days (Fig 3A). This resulted in a 3 to 3.5-fold increase in CaSR protein expression (Fig 3B). In subsequent experiments, over-expressing cells (denoted HAoSMC^CaSR+^) were compared with cells transfected with control plasmid (denoted HAoSMC^control^).

Transfected cells were cultured for 7 days under static conditions. HAoSMC^control^ showed a calcifying response (Fig 3C and 3D) identical to untransfected static HAoSMC (Fig 1A and 1B). In *Ca5* HAoSMC, over-expression reduced the calcification area from 70% (95%CI 55–85) to 42% (95%CI 25.5–58, p = 0.007, Fig 3C and 3D), a reduction similar in magnitude to that observed with cyclic strain (Fig 1A and 1B). Significant reductions also occurred with *Gd* HAoSMC^CaSR+^ (1.5%, 95%CI 0.3–2.6 versus HAoSMC^control^ 5.4%, 95%CI 2.5–8.2, p = 0.007) and *Gd+Ca2* HAoSMC^CaSR+^ (1.8%, 95%CI 0.8–2.9 versus HAoSMC^control^ 7.0, 95%CI 3.4–10.5, p = 0.004).

The effects on OC and ALP concentrations of CaSR over-expression were opposite to those observed with CaSR silencing. Under control conditions, OC concentrations were 10 (95%CI 6.4–13.6) ng/ml/mg in HAoSMC^CaSR+^, compared to 16.8 (95%CI 11.8–21.8) ng/ml/mg in HAoSMC^control^ (p = 0.01). With the addition of any of the CaSR agonist combinations, OC remained significantly lower in HAoSMC^CaSR+^ compared to HAoSMC^control^ (*Ca2* 10.1 (7.3–12.8) vs 18.3 (10.4–26.2), p = 0.02; *Ca5* 14.6 (9–20.1) vs 24.5 (17.7–31.2), p = 0.01; *Gd* 12.6 (8.3–16.9) vs 20.7 (14–27.4), p = 0.02; *Gd+Ca2* 13.4 (10.2–16.7) vs 24.9 (18.9–30.9), p = 0.002, Fig 3E). ALP activity was lower with CaSR over-expression compared to controls (Fig 3F), and this reduction was significant in the case of *Ca5* (56.3 (44.5–68.1) vs 72 (61–83), p = 0.02).

### Calcimimetic R568 inhibits calcium deposition by HAoSMC

**Fig 4:**
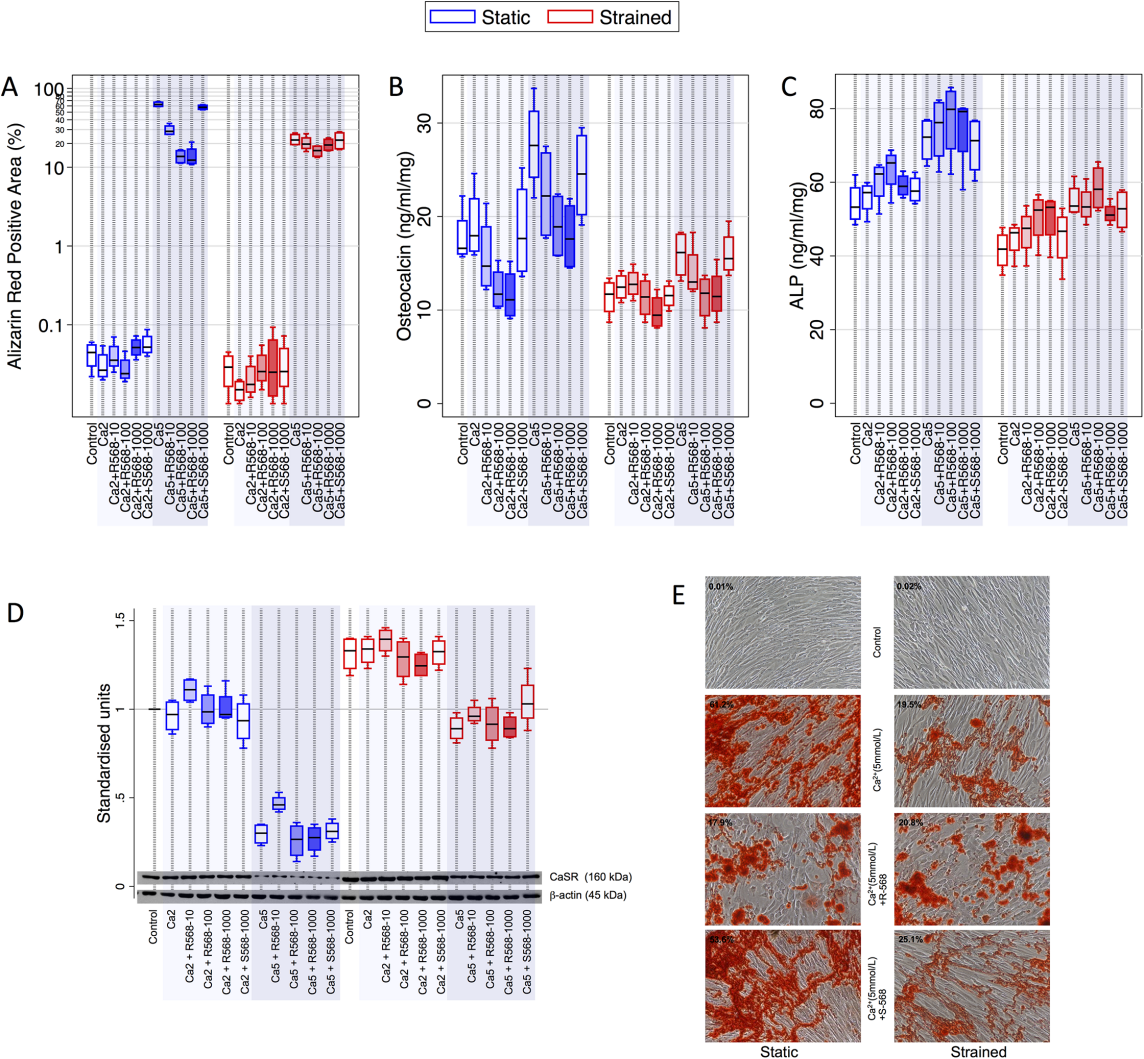
Effect of calcimimetic R568 and extracellular calcium on HAoSMC calcification and phenotype. Shading of box plots represents increasing concentrations (10, 100 and 1000 nmol/L) of R568. **A)** Calcium deposition (measured by alizarin red, n = 4 per condition, logarithmic y-axis) was reduced by R568 in a dose-dependent manner in the presence of *Ca5*, whereas the inactive enantiomer S568 had no effect on calcification. The effect of R568 was less pronounced under cyclic strain, where basal calcification is lower. **E)** Representative photographs of cells stained with alizarin red (magnification x100). **B)** OC expression (n = 4 per condition) was reduced by increasing concentrations of R568 in the presence of both *Ca2* and *Ca5*. These effects were also evident under cyclic strain, although less pronounced. **C)** ALP activity (n = 4 per condition) was not significantly influenced by R568. **D)** CaSR expression (n = 4 per condition) was not convincingly altered by exposure to R568, alhough low doses (10nmol/L) appeared to attenuate *Ca5-*induced reduction in CaSR expression in static HAoSMC. OC–osteocalcin; ALP–alkaline phosphatase; CaSR–calcium-sensing receptor.

Since calcium and gadolinium are not only CaSR agonists but may directly precipitate calcification, we next assessed the effects of a selective CaSR agonist (R568), which does not directly participate in calcium crystal formation, on HAoSMC. Cells were cultured under *control*, *Ca2* and *Ca5* conditions with the addition of varying concentrations of R568 or its inactive enantiomer, S568, both static and under cyclic strain (Fig 4).

As in previous experiments, *control* and *Ca2* HAoSMC and sHAoSMC did not show any evidence of calcification after 7 day incubation, and no calcification was evident in *Ca2-R568* or *Ca2-S568* HAoSMC and sHAoSMC (Fig 4A and 4E). As seen previously, static *Ca5* HAoSMC exhibited 63% (55.9–70.1%) alizarin red staining. The addition of R568 markedly reduced calcification in a dose- dependent manner with maximal suppression at a concentration of 100nmol/L R568 [29% (22.4–37.1%, p<0.0001), 10nmol, 13.8% (9.4–18.2%, p<0.0001) and 14.1 (6.7–21.4%, p<0.0001) with 1000nmol/l]. In contrast, calcification was not reduced by 1000nmol/L S568 (57.7%, 51.2–64.2%, p = 0.13).

Again, we observed a reduction in calcification with cyclic strain. In these less calcified sHAoSMCs, although calcification was reduced by exposure to R568, this effect was more subtle and only reached significance with 100nmol/L R568 (p = 0.03).

We next considered the effect of R568 on OC expression and ALP activity. In static HAoSMC, OC expression was reduced by increasing concentrations of R568, and this reduction was significant with 100nmol/L and 1000nmol/L in *Ca2* (p = 0.02 and p = 0.02 respectively) and *Ca5* HAoSMC (p = 0.03 and p = 0.02 respectively), as shown in Fig 4B. Under cyclic strain, where the overall OC expression is lower, we observed a similar trend. However, the reduction in OC expression only reached significance in *Ca5 s*HAoSMC at 100nmol R568. S568 did not alter OC expression under any conditions. In contrast to OC, ALP activity was not altered by R568 (Fig 4C).

We next sought to determine whether exposure of HAoSMC to R568 altered CaSR protein expression. In static *Ca5-R568* HAoSMC, low concentrations (10nmol/L) of R568 were associated with a small increase in CaSR content compared to *Ca5* HAoSMC, though this effect was not evident at higher doses of R568 or in *Ca5-R568* sHAoSMC. Overall therefore, CaSR protein expression does not appear to be altered by R568 or S568 (Fig 4D).

### Effects of R568 on arterial explants from healthy volunteers and CKD patients

**Fig 5:**
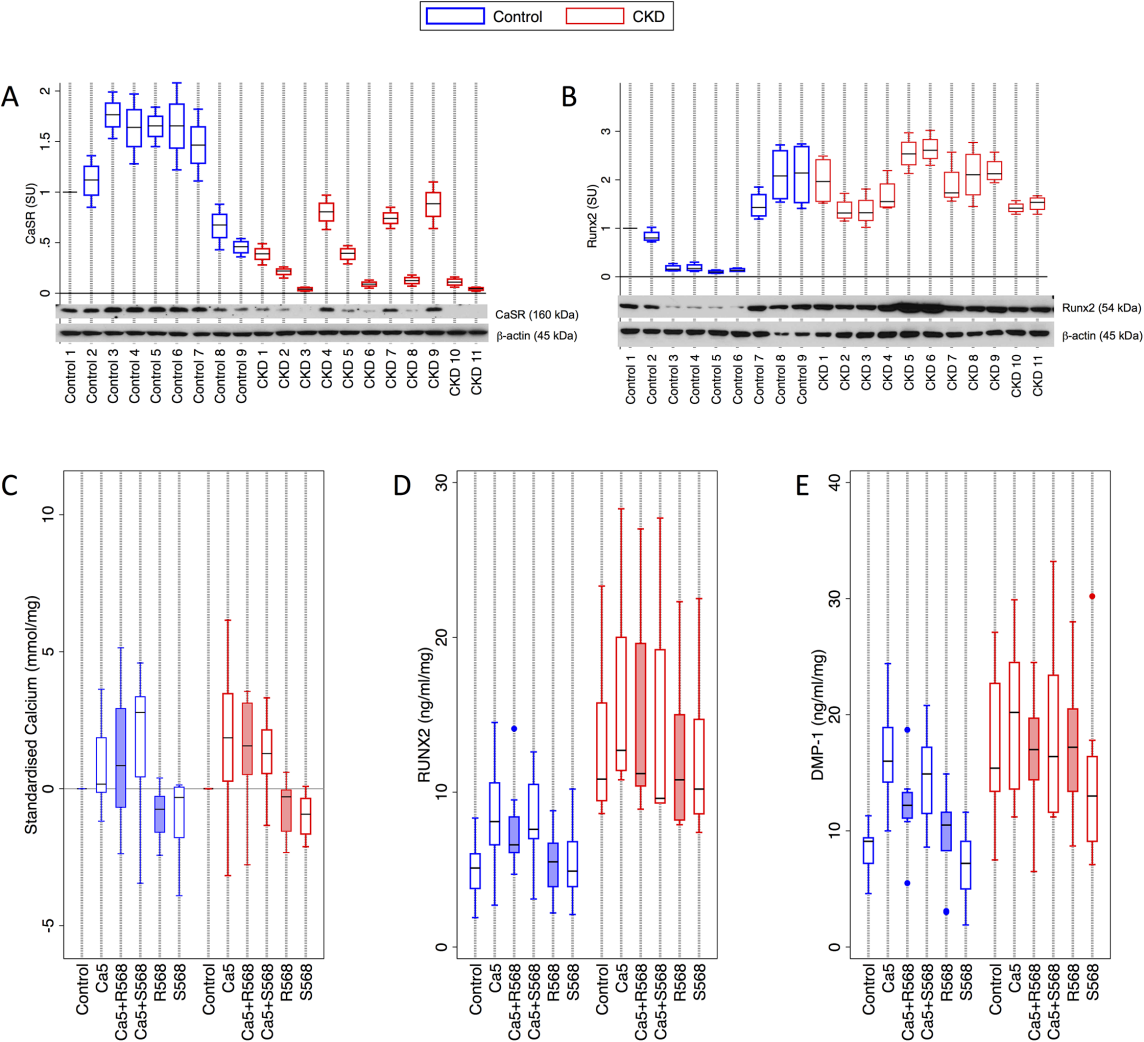
Effect of R568 and CaSR agonists on calcium deposition and osteogenic markers in arterial explants. Medium sized arterial explants from healthy individuals donating a kidney (control, n = 9) and patients with CKD (n = 11) undergoing a kidney transplant were cultured for 7 days. Shaded boxes represent exposure to R568 (100nmol/L). **A)** CaSR and **B)** Runx2 expression in Control and CKD artery (n = 4 per sample). CaSR expression was significantly lower in CKD patients (p = 0.0003), whereas Runx2 was significantly higher (p = 0.008). **C)** Total artery calcium content was not significantly different between control and CKD patients, and not significantly altered by R568. Expression of **D)** Runx2 (p = 0.0003) and **E)** DMP–1 (p = 0.0003) was significantly higher in CKD arteries compared to controls. R568 exposure did not significantly alter expression of these proteins. DMP–1 –dental matrix protein–1; CaSR–calcium-sensing receptor.

Given that these experiments demonstrated a role for the CaSR in the inhibition of calcification in HAoSMC, we next turned to human arterial explants to determine whether similar effects could be observed in intact artery. Clinical characteristics of patients donating medium sized arteries are detailed in Table 1. First, we determined the protein expression (standardised for protein concentration) of the CaSR and Runx2 in medium sized arteries from 9 healthy kidney donors and 11 kidney transplant recipients. Although variable, mean CaSR expression was 3.5-fold lower in CKD arteries than controls (p = 0.003) (Fig 5A), whereas mean Runx2 expression was more than 2-fold higher (p = 0.008, Fig 5B).

We next incubated arterial explants from this patient cohort *ex vivo* with *control* and *Ca5* media, with or without the addition of 100nmol/L R568 (or S568), for 7 days. Each arterial section was divided into subsections to allow intra-individual comparison across experimental conditions. From these samples, we determined total artery calcium content (expressed as calcium protein ratio), Runx2 and DMP–1 concentrations as markers of osteochondrogenic transformation.

In artery from CKD patients, we did not note a significant difference in basal artery calcium content. When assessing artery content across all conditions using anova however, artery calcium was higher in CKD samples (p = 0.038). In the absence of *Ca5*, neither R568 nor S568 exposure altered calcium content significantly compared with controls, both in CKD and control artery (Fig 5C). In the presence of *Ca5*, although artery calcium content tended to be higher, this was not statistically significant and calcium content was not modulated by R568 or S568 (Fig 5C).

We next assessed the extent of arterial smooth muscle cell ostechondrogenic transformation in these samples by measuring artery Runx2 and DMP–1 protein expression. Overall, Runx2 (p<0.0001) (Fig 5D) and DMP–1 (p = 0.0001) (Fig 5E) were higher in CKD artery compared to controls. Exposure of control arteries to *Ca5* resulted in increased expression of both Runx2 (p = 0.0003) and DMP–1 (p = 0.0003). Exposure of control *Ca5* arteries to R568 significantly reduced DMP–1 expression (p = 0.03), but did not alter Runx2 expression (p = 0.5) (Fig 5C and 5D). In CKD artery, where basal protein concentrations of both Runx2 and DMP–1 protein were significantly higher, modulation of extracellular calcium and the addition of R568 did not significantly alter Runx2 and DMP–1.

## Discussion

Accumulating evidence suggests that the CaSR is functionally expressed in the vasculature and may play a major role in the pathogenesis of arterial calcification, which accompanies the development of atherosclerosis, arteriosclerosis and hypertension [5,30,33,34]. Here, we confirmed previous reports demonstrating that HAoSMC undergo calcification in response to high extracellular concentrations of the CaSR agonists [23]. We show for the first time that the extent of calcification and osteochondrogenic transformation is inversely associated with SMC CaSR expression; that cyclical mechanical strain preserves CaSR expression under pro-calcific conditions, and inhibits calcification and osteochondrogenic transformation; and that the calcimimetic R568 inhibits calcification and osteogenic transformation by maintaining CaSR expression.

The arterial tree is continually subjected to mechanical forces including cyclic stretch resulting from pulsatile blood pressure [35]. Importantly, vascular calcification often occurs in regions with an altered mechanical environment such as native heart valves and vascular lesions, suggesting a role for mechanical stimuli in calcification [36]. Recent findings suggest that mechanical strain can activate cation channels, G proteins and G protein-coupled receptors, which may serve as non-specific mechanosensors [37]. Our findings indicate that mechanical stretch may also activate the CaSR in HAoSMC.

We found that CaSR agonists induced a pronounced down-regulation of CaSR expression, transformation to osteogenic phenotype and a concomitant increase in VSMC calcification. Reduced CaSR expression was previously noted in bovine vascular SMC [23], and similar responses have been observed in other cell types expressing the CaSR. In murine osteoblasts, Dvorak et al [38] demonstrated increased mineralised nodule formation after exposure to Ca^2+^ and Gd^3+^. In the chondrogenic cell line C5.18, exposure to between 2 and 4mmol/L Ca^2+^ resulted in increased extracellular matrix mineralisation and OC expression [39].

Calcification and osteogenic transformation was promoted by CaSR knockdown and inhibited by over-expression, demonstrating that the presence of a functional CaSR was directly protective against the development of a calcifying phenotype. The central role of the CaSR is consistent with the findings of Alam et al [23], who reported that over-expression of dominant-negative CaSR enhanced mineral deposition by VSMC, while calcimimetic R568 suppressed Ca^2+^ and phosphate-induced mineralisation. In a chondrocytic cell line, Chang and colleagues similarly showed that OC expression and mineralisation in response to CaSR agonists were inhibited by expression of wild-type CaSR cDNA and promoted in cells expressing a signalling-defective CaSR mutant [39].

Introduction of cyclic strain not only maintained CaSR expression, but also significantly inhibited high calcium-induced down-regulation of the CaSR and associated increased calcium deposition. Given that cyclic strain mimics conditions in healthy human artery, which is exposed to pulsatile stress, these findings suggest that physiological strain *in vivo* might prevent arterial calcification and phenotypic transformation through its effects on CaSR expression. Our findings are supported by the findings of Nikolovski et al [40] who demonstrated that in rat aortic SMC, long-term mechanical strain down-regulated the expression of bone-associated genes and significantly reduced calcium deposition. A precedent also exists in osteoblast-like cells, where mechanical stretch limits osteoblast maturation, OC expression and ALP activity, and matrix mineralisation [41,42].

Consistent with the above observations, we found that the calcimimetic R568 protected against the calcium-induced decrease in CaSR expression and partially restored its expression in static calcifying cultures of HAoSMC. This was accompanied by a dramatic reduction in OC production and calcium deposition. The protective effects of R568 were less pronounced in strained HAoSMC cultures. This may be attributed to the effect of strain itself on CaSR expression, calcification and phenotype, where CaSR stimulation may already be near-maximal.

These findings add to a growing body of evidence indicating that calcimimetics protect against vascular calcification, mediated through the CaSR. Ivanovski et al [28] demonstrated inhibition of arterial calcification by R568 in a murine model of atherosclerosis, and showed that this protective effect was abolished by siRNA CaSR silencing. Further, Henaut et al [24] subsequently showed that exposure of HAoSMC to R568 or AMG641 induced CaSR synthesis and transport from the endoplasmic reticulum to the plasma membrane. Conversely, calcification of VSMC has been associated with a loss of CaSR expression [23], and we have previously demonstrated reduced CaSR expression in calcified human arteries [21].

Taken together, these data suggest that in settings where vascular calcification is highly prevalent, treatment with calcimimetics holds the potential to reduce or prevent vascular calcification. This is particularly pertinent to CKD, where calcimimetics may also have indirect effects on calcification through its effects on biochemical parameters such as PTH, calcium and phosphate concentrations [5,43].

In human arterial explants from CKD patients and controls, calcium content was higher in CKD arteries than controls, but not significantly influenced by co-incubation with high calcium or R568. Runx2 expression in the tunica intima and media of calcified CKD arteries was previously demonstrated by Moe et al [44] and confirmed by others [45,46]. DMP–1 is a marker of mineralising osteocytes [47,48], and is up-regulated in animal models of aortic medial calcification [49]. We found that CKD arteries not only exhibited low basal CaSR and increased Runx2 expression as reported previously [50], but also demonstrated increased DMP–1 expression, indicative of a mineralising phenotype. Further, we found that exposure of arterial explants to high calcium concentrations resulted in significant increases in both Runx2 and DMP–1 expression. This increment in expression was not evident in CKD arteries, possibly because of pre-existing near-maximal induction of osteogenic pathways. Similarly, R568 reduced DMP–1 and Runx–2 expression in control arterial explants. This was not significant in CKD explants, where osteogenic phenotypic transformation had already occurred. Together, these data add to the growing body of evidence that CKD is associated with arterial osteogenic phenotypic transformation, and the marked inhibition of DMP–1 expression and lowering of Runx2 by R568 in arterial explants support a role for calcimimetics in preventing arterial calcification. Lopez et al [51] recently demonstrated reductions in aortic calcium and phosphorous deposition and increased expression of the calcification inhibitor matrix Gla protein in healthy and uremic rats exposed to the calcimimetic AMG641, suggesting one potential mechanism by which calcimimetics could prevent vascular calcification [25].

In conclusion, we have demonstrated an inverse association between VSMC CaSR expression and calcification. We have shown that an osteogenic phenotype can be directly modified by modulating CaSR expression and found that CaSR expression is preserved by cyclic strain. Further, our finding of reduced calcification and phenotypic transformation by the calcimimetic R568 supports the use of calcimimetics to reduce or prevent arterial calcification. Accelerated calcium deposition in arterial explants from CKD is accompanied by reduced CaSR expression and further osteocyte transformation, a process partially inhibited by R568. Collectively, our data suggest that expression of a functional CaSR may prevent transformation towards a calcifying VSMC phenotype and, therefore, protect against calcification.

## Supporting Information

S1 Fig Expression of smooth muscle α-actin and CaSR in HAoSMC cultured under a cyclic strain. HAoSMC were cultured under a cyclic biaxial strain for up to 14 days. Cell lysates were separated by 10% SDS-PAGE and blotted with anti-α-actin (A), anti-CaSR (B) and anti-β-actin (A, B). Densitometry was performed using Image J Analysis software. The data are presented as mean±SD (n = 4) with antigen expression shown as standardised fold increase/decrease from control. *p<0.05, **p<0.01 *vs* 1 day static culture.
(PDF)
