# Supplementary material for: Arterial Expression of the Calcium-Sensing Receptor Is Maintained by Physiological Pulsation and Protects against Calcification
Source: PLoS One. 2015 Oct 5;10(10):e0138833. doi: 10.1371/journal.pone.0138833 (PMC4593585; doi:10.1371/journal.pone.0138833)
Supplement: S1 Fig — HAoSMC were cultured under a cyclic biaxial strain for up to 14 days. Cell lysates were separated by 10% SDS-PAGE and blotted with anti-α-actin (A), anti-CaSR (B) and anti-β-actin (A, B). Densitometry was performed using Image J Analysis software. The data are presented as mean±SD (n = 4) with antigen expression shown as standardised fold increase/decrease from control. *p<0.05, **p<0.01 vs 1 day static culture. (PDF) [file pone.0138833.s001.pdf]

SUPPLEMENTAL MATERIAL

Detailed methods

Cell culture

Primary cultures of HAoSMC (PromoCell, Germany) were maintained in complete VSMC growth medium 2 containing 5% FCS, 0.5 ng/ml epidermal growth factor, 2.0 ng/ml basic fibroblast growth factor, and 5g/ml insulin (PromoCell). The cells were grown under 5% CO_2 at 37°C in medium renewed every 3 days. Confluent cells were detached by trypsin/EDTA and subcultured with a split ratio 1:2. HAoSMC in the experiments were used between passages 2 and 5. Cells were plated at 2×10^5 cells/well of a 6-well plate and grown in DMEM/F-12 (1:1) with 5% FCS. For cyclic strain culture, cells were plated into 6-well collagen 1 coated Bioflex plates (Flexcell, USA) and cultured under cyclic biaxial strain (7% stretch, 30 cycles/min) for up to 14 days using a Flexcell FX-4000 unit (Flexcell). For calcification experiments, cells were incubated for 7 days with 2 and 5mmol/L Ca^{2+}, 50μmol/L Gd^{3+} or with a combination of 2mmol/L Ca^{2+} and 50μmol/L Gd^{3+}. Alternatively, HAoSMC were treated with 2 and 5mmol/L Ca^{2+} in the presence of 10, 100, 1000nmol/L calcimimetic R-563, an allosteric activator of the CaSR, or 1000nmol/L S-568 (inactive enantiomer) (Amgen, USA). To facilitate mineralisation 5mmol/L β-glycerophosphate was added to all experiments where calcification was studied.

Culture of human arterial explants

Arterial explants (3-4mm) from 9 healthy individuals donating a kidney (control) and 11 patients with advanced CKD undergoing a renal transplant were cultured in complete VSMC growth medium 2 for 7 days and treated with 5mmol/L Ca^{2+} with or without 100nmol/L R-568 or 100nmol/L S-568.

Inhibition of CaSR expression by specific siRNA

To examine the functional role of the CaSR in HAoSMC, we used siRNA to knock down the level of CaSR expression (Santa Cruz Biotechnology). The CaSR siRNA is a pool of 3 target-specific 20-to25-nucleotide siRNAs designed to inhibit gene expression. The transfection of siRNA specifically targeted to the CaSR into HAoSMC was performed using Lipofectamine (Invitogen) according to the manufacturer’s protocol. Lipofectamine and siRNAs were diluted into OptiMEM medium (Invitro-gen). Diluted Lipofectamine lipids were mixed with diluted siRNAs and incubated for 30 min at room temperature for complex formation. Mixtures were further diluted in OptiMEM and added to each well so that the final concentration of siRNAs was 40 nmol/L. Control siRNA-A and Lipofectamine alone were used as negative controls. To maintain inhibition of the CaSR expression in longer-term cultures, transfection was repeated every 3 days. CaSR knockdown was monitored by Western blotting. Following transfection the cells were cultured for 7 days and treated with 2 and 5mmol/L Ca^{2+}, 50μmol/L Gd^{3+} or in combination.

Over-expression of the CaSR by pcDNA3.1CaSR+

For transfection, full length human CaSR was cloned into pcDNA3.1(+) containing hygromycin selection gene. Plasmid DNA was prepared using Qiagen plasmid midi kit (Qiagen). HAoSMC were seeded at 2×10^5 cells/well of 6-well plate, allowed to adhere overnight, and washed twice with OptiMEM medium (Invitrogen). Transfection of pcDNA3.1CaSR+ into HAoSMC was performed using Lipofectamine LTX with Plus Reagent (Invitogen) according to manufacturer's protocol. Lipofectamine and plasmid DNA were diluted into OptiMEM. Diluted lipofectamine lipids were mixed with diluted plasmid DNA and incubated for 30 min at room temperature for complex formation. Mixtures were further diluted in OptiMEM, carefully added to each well to make final concentration of 2μg/mL and incubated overnight. After 24 hour rest in complete medium, the cells were cultured for 72 hours in selection medium containing 100μg/mL hygromycin (Sigma). Control (empty) pcDNA3.1(+) and lipofectamine alone were used as negative controls. The effectiveness of transfection (CaSR overexpression) was monitored by RT-PCR and Western blotting. After transfection HAoSMC were cultured for 7 days and treated with 2 and 5mmol/L Ca^{2+}, 50μmol/L Gd^{3+} or in combination.

Analysis of the CaSR mRNA expression

Total RNA was isolated from HAoSMC lysates using RNA easy kit (Qiagen, UK) following the manufacturer's protocol. Arterial samples from patients were homogenised in liquid nitrogen and solubilised using the lysis buffer from the same kit. Reverse transcription of total RNA was carried out using a BioScript reverse transcriptase with random hexamers (Bioline, UK).

PCR amplification of CaSR cDNA was performed using the following primers: 5-TTCCGCAACACACCCATTGTCAAGG-3 and 5-GGATCCCGTGGAGCCTCCAAGGC-3 (1). PCR reactions (40µl) were set up using 1× reaction buffer, which contained 16mmol/L $(NH_4)_2SO_4$, 67mmol/L TrisHCl (pH 8.8), 0.01% Tween 20, 1.5 mmol/L MgCl2, 0.2 mmol/L of each dNTP, 0.5mol/L of each primer, and 1U of BIOTAQ DNA polymerase (Bioline). Amplification was performed using an initial denaturation step (95°C for 5 min) followed by 35cycles of 95°C (1 min), 58°C (1 min), and 72°C (1 min). In addition, a final elongation step of 72°C for 7 min was included. Control PCR was carried out using primers specific for 18S ribosomal RNA, producing a PCR product of 324 bp. RT-PCR products were separated on a 1% agarose gel. The presence of a 816-bp amplified product was indicative of positive PCR arising from the CaSR-related sequence in cDNA. Purified PCR products were verified by sequencing on anautomatic DNA sequencer (ABI, Warrington, UK) using the same primers as for the RT-PCR.

Western blot analysis

HAoSMC and arterial explants were treated with specified agonists and then harvested. The cells were washed three times with cold PBS, scraped, and solubilised in 80µl cold lysis buffer with freshly added protease inhibitor cocktail (Sigma, USA). Cell debris was pelleted by microcentrifugation at 10,000g for 10 min at 4°C. Aliquots of cell lysates containing 15µg protein were separated by SDS-PAGE and Western blotted with anti-human β-actin (New England Biolabs, UK), anti-human-α-SM-actin (Sigma) and anti-human Runx2 (Santa Cruz Biotechnology, USA).

For analysis of CaSR protein expression, the cells were solubilised on ice using lysis buffer containing 0.25 mol/L TrisHCl (pH 7.8), 0.5% Igepal, 5 mmol/L DTT, and freshly added protease inhibitor cocktail (Sigma). The lysates were then separated by 7% SDS-PAGE and Western blotted with a polyclonal anti-human CaSR (Binding Site, UK). Densitometry was performed using Image J Analysis software using blots from four independent experiments with final results normalised against β-actin.

Alizarin red staining

HAoSMC were rinsed with 0.9% NaCl solution (pH=7.4), stained with 1% alizarin red (pH=4.2) for 1 min and further rinsed twice with 0.9% NaCl. Stained cell cultures were photographed (×100) using Nikon Eclipse TS100 System. The percentage of alizarin red-positive areas was measured using Image J analysis software.

Analysis of osteoblast-like phenotype

OC production was assayed using N-MID OC ELISA (IDS, UK) following the manufacturer’s protocol. After treatment the cells were harvested, solubilised with RIPA lysis buffer and assayed using N-MID OC ELISA. ALP activity was measured using SensoLyte *p*-Nitrophenyl phosphate (*p*NPP) ALP assay kit (AnaSpec, USA) according to the manufacturer’s protocol. Following treatment the cells were scraped, solubilised and assayed. Runx2 and DMP-1 expression in arterial explants was quantified with ELISA kits (MyBioSource, USA). Arterial tissue was homogenised in liquid nitrogen, lysed and assayed for protein concentration. OC, ALP, Runx2 and DMP-1 expression (ng/ml) was normalised against protein concentration of the samples.

Calcium content measurement

Calcium was measured by a colorimetric assay using orthocresolphthalein complexone method developed by Schwartzenbach and modified by Connerty & Briggs, 1966 (2). Reagents were obtained from the Siemens Dimension RXL (Siemens Healthcare). Arterial explants were homogenised, lysed and assayed for protein concentration. The extracts were acidified with 50% HCl, mixed and allowed to stand for 20 minutes at room temperature. Tubes were briefly centrifuged before analysis, which was performed in NUNC microtitre plates (Perkin Elmer). The absorbance was read at 550nm using Victor3 plate reader (Perkin Elmer). Calcium content (mmol/L) was normalised against protein concentration of the samples.

Figure 1

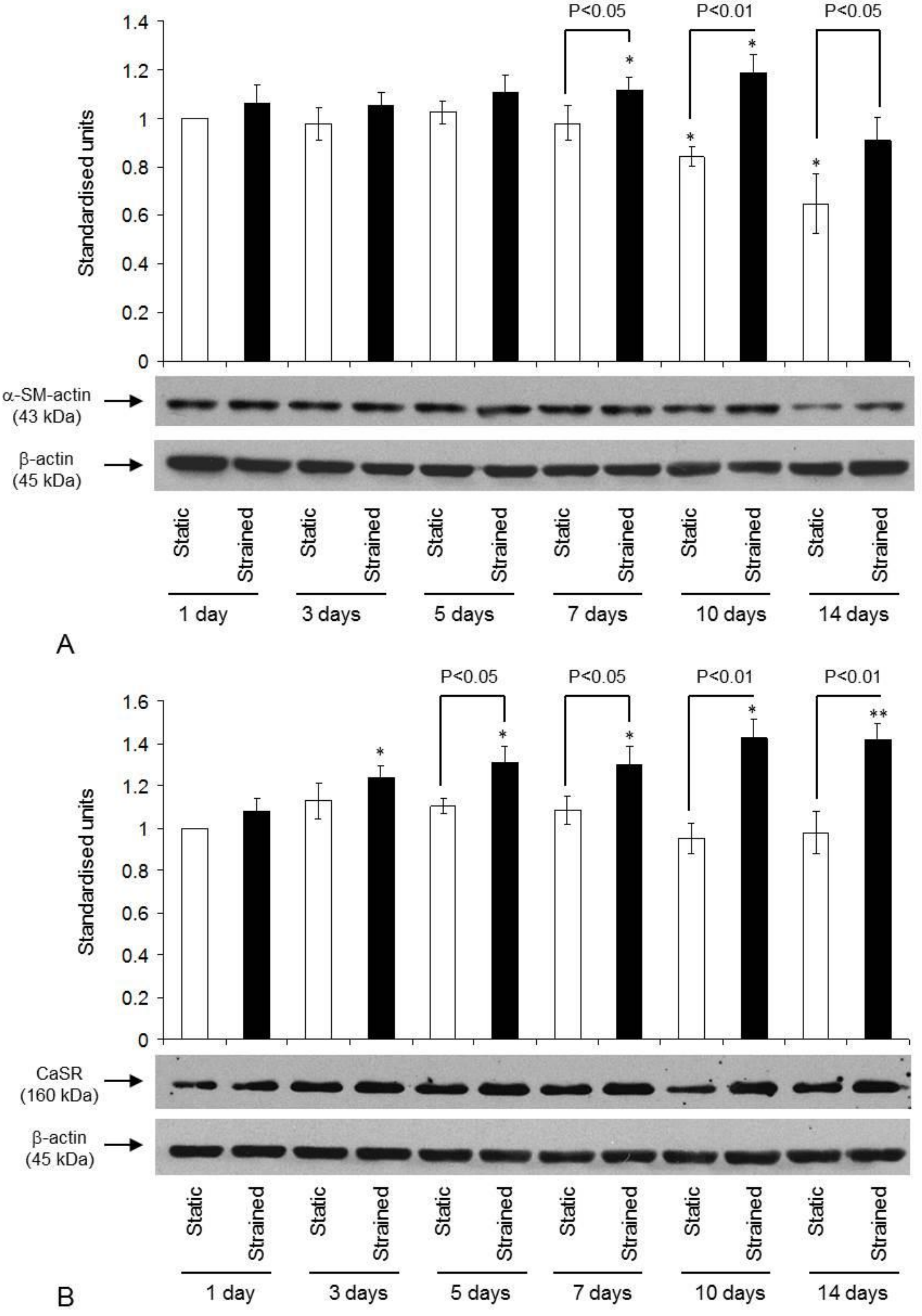

S1 Fig. Expression of smooth muscle α-actin and CaSR in HAoSMC cultured under a cyclic strain. HAoSMC were cultured under a cyclic biaxial strain for up to 14 days. Cell lysates were separated by 10% SDS-PAGE and blotted with anti-α-actin (A), anti-CaSR (B) and anti-β-actin (A, B). Densitometry was performed using Image J Analysis software. The data are presented as mean ±

SD (n=4) with antigen expression shown as standardised fold increase/decrease from control. *p<0.05, **p<0.01 *vs* 1 day static culture.

References

1. Molostvov G, James S, Fletcher S, Bennett J, Lehnert H, Bland R, Zehnder D. Extracellular calcium-sensing receptor is functionally expressed in human artery. Am J Physiol Renal Physiol. 2007;293: F946-955.
2. Connerty HV, Briggs AR. Determination of serum calcium by means of orthocresolphthalein complexone. Am J Clin Pathol. 1966;45: 290-296.
